# Quantitative Genetics in the Genomics Era

**DOI:** 10.2174/138920212800543110

**Published:** 2012-05

**Authors:** William G. Hill

**Affiliations:** Institute of Evolutionary Biology, School of Biological Sciences, University of Edinburgh, West Mains Road, Edinburgh, EH9 3JT, UK

**Keywords:** Complex traits, evolution, heritability, genetic variance, genome wide association, QTL, selection.

## Abstract

The genetic analysis of quantitative or complex traits has been based mainly on statistical quantities such as genetic variances and heritability. These analyses continue to be developed, for example in studies of natural populations. Genomic methods are having an impact on progress and prospects. Actual relationships of individuals can be estimated enabling novel quantitative analyses. Increasing precision of linkage mapping is feasible with dense marker panels and designed stocks allowing multiple generations of recombination, and large SNP panels enable the use of genome wide association analysis utilising historical recombination. Whilst such analyses are identifying many loci for disease genes and traits such as height, typically each individually contributes a small amount of the variation. Only by fitting all SNPs without regard to significance can a high proportion be accounted for, so a classical polygenic model with near infinitesimally small effects remains a useful one. Theory indicates that a high proportion of variants will have low minor allele frequency, making detection difficult. Genomic selection, based on simultaneously fitting very dense markers and incorporating these with phenotypic data in breeding value prediction is revolutionising breeding programmes in agriculture and has a major potential role in human disease prediction.

## INTRODUCTION

The explanation of how traits could be both continuously distributed and yet depend on particulate inheritance was resolved within a few years of the Mendelian rediscovery. The model of effects of multiple genetic loci and the environment contributing to the phenotype accounts straightforwardly for the typically Gaussian phenotypic distribution of continuous traits, the observed correlations among relatives, inbreeding depression and heterosis, continuing responses to artificial selection, and evolutionary change. Analyses and predictions have been based almost entirely on phenotypic observations and their interpretation in terms of measures such as components of genetic variance and covariances, heritability, dominance, and changes in frequency of many genes [1-3]. Many of the principles and ideas developed can also be used more broadly to include pedigree and phenotypic data on all complex traits, such as discrete value traits describing presence or absence of disease that do not show simple Mendelian inheritance but may be described by, for example, a threshold model, or continuous traits such as survival time that do not have Gaussian distributions.

Research in recent decades has provided both direct and indirect evidence of the location and effects of individual loci affecting quantitative traits and, for a limited number of loci, knowledge of the causative change in the DNA. Progress has, however, been restricted by many factors. These include the inability to disentangle the effects of closely linked genes through limitations in amount and type of data, the difficulty of following the metabolic trail from a base substitution to a change in the trait, and the potential interactions among genes at the metabolic and phenotypic level. The rapid advance in genomic methods and other high throughput ‘-omic’ methods has both fuelled the recent developments and provides the opportunity for more. The advances are not restricted just to mapping and analysing individual loci but involve an increasing integration of molecular and statistical methods. With dense genetic markers used throughout the whole genome, the genetic covariances among relatives can be partitioned among genomic regions and incorporated into classical statistical analyses developed for phenotypic data to predict offspring performance in plant and animal improvement programmes and in the prevention of human disease. Nevertheless there is still a long way to go.

Quantitative genetic understanding and application are also being informed by progress in the analysis, interpretation and utilisation of solely phenotypic data, facilitated by developments in statistical methods and computing power and by the availability of pedigree information in natural populations from long term records or genetic markers. Lest it be thought that, in the genomic era, quantitative genetic methods have been replaced rather than supplemented, I shall discuss these first. 

This review is from the perspective of a quantitative rather than molecular geneticist, concerned more with understanding the output of processes affect the phenotype rather than on what the processes are. It is not intended to be comprehensive. There is an expanding compendium in the work of Walsh and Lynch [3], and many recent reviews of some or most of the topics [e.g. 4-7]. Other topics are discussed in more detail elsewhere in this volume; for example the genetic analysis of complex traits with discrete phenotypes such as disease risk is a major focus of human genetics [8] but important in many other situations. Genomic approaches have many applications in population genetics which in turn inform the analysis, interpretation and utilisation of quantitative genetic variation. These include inferences about population structure, such as genetic distance, inbreeding level and effective size in both natural and domesticated species. Although these essentially population genetic analyses at the level of individual loci impact in turn on predictions of, for example, maintenance of variation and opportunities for long term genetic change for polygenic traits, I shall not pursue them further here except in some situations where they relate directly to the architecture of quantitative genetic variation. 

## ANALYSIS OF VARIATION AND COVARIATION

The basic descriptors of the phenotypic variability of quantitative traits have not changed greatly over recent decades, subsequent to the adoption of the ‘animal model’, led by Henderson, which generalises the description of the variances and covariances of all members of the population [2, 9-11]. Although widely used in the livestock context, it applies to all species, and is merely a linear model of each of the fixed effects (e.g. years) and random genetic and non-genetic effects contributing to each individual’s phenotype for one or more traits, combined with a series of matrices defining the covariances of effects of individuals in the population. For example the covariance of breeding values is the product of the relationship matrix (usually denoted **A**) and the additive genetic variance of the trait or covariance of a pair of traits. Although usually used in the context of the infinitesimal model of very many unlinked additive genes of small effect, it can also include discrete effects due to the genotype at individual loci. 

In situations typical of most natural populations or livestock the data are highly unbalanced, and methods for parameter estimation have been developed that have become more powerful as computing speed has increased. Currently most use is made of residual/restricted maximum likelihood (REML), facilitated by the availability of general packages such as ASREML ([12], http://www.vsni.co.uk/downloads/asreml/)*.* Bayesian methods are increasingly being employed, enabled by Markov Chain Monte Carlo Methods (MCMC) methods [9], and general packages are becoming available (e.g. Bugs or Jags, http://www-ice.iarc.fr/~martyn/software/jags/). The Bayesian methods provide posterior distributions of parameters rather than just modes as in REML, and an integrated estimation, prediction and model selection machinery. The accompanying MCMC methods are readily generalised to deal with non-normal data, for example where an unspecified number of QTL can be fitted simultaneously. Nevertheless, the Bayesian methods make much greater computational demands. 

The appeal of the animal model lies in its inclusivity, conceptual simplicity and flexibility: basically the phenotype is expressed as the sum of fixed effects, such as year, and random effects such as breeding value (i.e. sum of additive genetic effects), maternal genetic and common sib environment. The random effects are defined by their variances and their covariances which, for breeding values, are proportional to the relationship among each pairs of individuals. The data may be balanced or unbalanced, there may be single or multiple traits, and individuals that have records and those that do not are both included in the relationship matrices. Genotype x environment and age effects may appear as fixed and/or random effects, for example using random regressions to define different genotypic effects of age. Recent developments include the incorporation of competition effects, following ideas put forward many decades ago but only recently incorporated into the standard framework in a form analogous to maternal effects. An individual’s phenotype, weight, for example, is defined in terms of its own direct genetic and non-genetic effects and of indirect influences on it from, say, livestock pen-mates or adjacent trees, expressed as the sum of the competitive effects of all its contemporaries [13, 14].

Homogeneity of variance, following any necessary transformation, is a basic assumption in many analyses in quantitative genetics. There has been recent interest in assuming genetic heterogeneity in the environmental variance, i.e. that the variance of phenotype given breeding value depends on the genotype, which is relevant to the evolution of variability and to breeding opportunities to change product consistency. The expected variance of each genotype is expressed in terms of genetic effects that have in turn a covariance structure among individuals proportional to the relationship matrix [15, 16]. Analysis of data fitting such models has been developed using both Bayesian methods and others that are cruder but computationally less demanding (reviewed in [17]). Whilst a small, but significant, genetic variation in the environmental variance has usually been found, as yet there is little understanding of the causative effects. 

Analysis of the animal model using REML, for example, provides predictions of the breeding values of individuals that have records and of their relatives as yet without records or indeed unborn. Similarly, parameter estimates obtained from a REML analysis can subsequently be utilised in best linear unbiased prediction (BLUP) of breeding values with much less computing demands. The animal model provides what has become the classical framework for animal breeding using quantitative data which, as discussed later, is in turn being supplemented by genomic information. 

### Quantitative Genetic Analysis in Natural Populations

The study of the inheritance and evolution of quantitative traits in natural populations has been handicapped by the absence of long term pedigrees and often of much information on the ecology or population structure of species otherwise suitable for study. For example, little is known about the life history of *Drosophila melanogaster* in the wild and on the relation between traits in natural populations and, for example, breeding success. Analyses in laboratory populations based on imposed artificial selection or of natural selection in a population cage are unlikely to account for all the evolutionary forces acting in nature on fitness and contributions to it of individual traits. 

In recent decades long term recording programmes have been established in fully or partially closed wild populations spanning multiple generations, for example in blue tits, sheep and deer. In these, pedigrees are established by identifying individuals at birth with their parents, if necessary supplemented by genetic markers to identify father, and records taken of multiple traits throughout the animals’ life. The animal model enables the data on what is an inevitably complex pedigree structure to be handled and, in principle, estimates to be obtained of both parameters of quantitative traits, such as genetic variances and inbreeding depression effects, and of the selection associated with each trait [3,18]; for some examples see Proc. R Soc. B, 2008, 275, 593-750. Whilst the natural selection is on fitness, the analysis can enable the selection gradient, the partial regression of the trait on fitness as measured by breeding success on each trait, to be assessed. Because the analysis provides predicted breeding values for each cohort relative to the base population (in this case start of recording), genetic trends can be estimated and compared with expectations or used to estimate environmental change [19]. 

A critical assumption in such analyses is that all the information on which selection decisions have been made is included in the data set. This may be reasonable in a breeding programme or laboratory experiment providing that a multi-trait analysis is undertaken, but may not be completely met as natural selection cannot be avoided completely: for example there may be differential survival prior to birth or first recording and consequent bias in estimates [20]. In studies of natural populations where the aim may be to understand their evolution in terms of what selection has actually occurred and on what traits and on how much selection response has occurred as a consequence, the assumptions become more critical. 

## INCORPORATION OF GENOMIC INFORMATION IN QUANTITATIVE GENETIC ANALYSES

### Inferring and Using Pedigree Relationship 

In many analyses of natural populations adequate pedigree information remains a limitation: the dam may be known from recording at birth or hatch but not the sire, or there may be extra-pair mating. There are also equivalent circumstances in breeding programmes, for example with multiple sire mating pens in poultry. Identification of more distant relatives may also be desired, for example to obtain estimates of parameters from covariances of relatives which do not share any common environment, or to identify the ancestry of non-pedigreed individuals low in a multiplication pyramid found to have commercially desirable properties such as extreme leanness or disease resistance. 

Whilst micro-satellite markers have mainly been used, with SNP panels it is becoming possible to use higher density markers to establish more distant and complex relationships. Pemberton [21] discusses methods and principles for identifying relationship and recently Powell *et al*. [22] suggested using as a reference point the current population rather than an ancestral one that depends on depth of pedigree. Software packages are available for parentage identification that can allow for genotyping errors, for example CERVUS ([23]; http://www.fieldgenetics.com/pages/aboutCervus_Using.jsp,) and COLONY ([24]; http://www.zsl.org/science/research/software/colony,1154,AR.html). 

### Utilisation of Actual Relationship and Inbreeding

Pedigree relationship describes the expected proportion of genes shared by relatives and similarly for pedigree inbreeding coefficient. Due to Mendelian segregation and linkage, the actual (or realised) proportion of genome shared identical by descent differs by chance from pedigree expectation (other than for offspring and parent). For example, for human full sibs the standard deviation of actual relationship is approximately 3.9% about the mean of 50%; and for more distant relatives, its coefficient of variation rises rapidly: for example for second cousins, the mean is 3.12% and SD 1.20% [25].

Genomic methods enable the actual sharing to be estimated from the genotypes at individual loci regardless of location in the genome. These include PLINK ([26]; http://pngu.mgh.harvard.edu/~purcell/plink/) and an alternative algorithm to remove bias by incorporating sampling error dependent on the number of loci [27]. Alternatively the actual sharing of genomes along the chromosome can be identified with programs such as BEAGLE ([28], http://faculty.washington.edu/browning/beagle/beagle.html). Inbreeding coefficients can be estimated similarly.

Actual relationship can be employed in analysis of quantitative genetic data. Thus common environmental effects of human full sibs can be eliminated by a regression of the variance between them in the trait on the proportion of genome shared by the pair [29, 30]. The estimate of heritability of height in the larger study [30] was 86%, very similar to those obtained previously by conventional between-family methods such as comparing correlations of MZ and DZ twins. Further, the estimate of dominance variance was non-significant, and an analysis fitting chromosomes one at a time as ordinary or partial variables gave similar values, indicating there was little epistasis of genes on different chromosomes for human height. There was a strong linear relationship between chromosome length and variance explained. Similar analyses could be done to estimate effects of inbreeding by regressing performance on actual identity by descent within families. 

In animal model analyses within populations the pedigree relationship or inbreeding coefficient can be replaced by the actual relationship; which should increase precision and also enables direct calculations for pairs of distant relatives where pedigree is not known but becomes irrelevant. As discussed later, actual relationship, computed from dense markers, is also used in breeding value prediction, and termed the genomic relationship matrix.

## THE GENETIC ARCHITECTURE OF QUANTITATIVE TRAITS - APPROACHES

Many questions in quantitative genetics, for example ‘what is the predicted response to selection in the next generation’ can be answered from measures such as heritability without any knowledge either of the underlying quantitative aspects of the genetic architecture, such as the number of genes and the distribution of their effects on the trait, their interactions with other genes and their frequency, or of more basic factors such as how do the trait genes act and how are they controlled. These are of course major issues, and in this discussion I shall concentrate attention on the first of these unknowns, the effects at the level of the trait and how this is being informed by genomic methods, rather than mechanisms of action.

A direct approach is to use random mutagenesis. Insertional mutagenesis is particularly powerful in that it leaves a signal such that the target gene can be mapped directly. It has, for example, been used very successfully by Mackay and collaborators [e.g. 31] to identify genes affecting many quantitative traits and to estimate their direct and pleiotropic effects. Mutational studies do not, however, necessarily identify genes that are contributing to population differences or to standing variation within a population or species. 

### Linkage Mapping

Until large numbers of microsatellite markers became available, mapping of QTL was essentially impossible except in some laboratory species. Subsequently, and with the introduction of interval mapping by maximum likelihood [32] or regression [33], extensive resources have been put into QTL mapping, initially using inbred line crosses or backcrosses but then developed to include composite mapping, fitting multiple loci, and family analysis in random mating populations [2, 34]. Package software programs are available, e.g. QTL Cartographer (http://statgen.ncsu.edu/qtlcart/WQTLCart.htm) and GridQTL (http://www.gridqtl.org.uk/index.htm).

The major limitation of linkage studies using F2s or backcrosses is the inability to do fine scale mapping. Insufficient density of markers to detect recombination was initially a limitation, now it is just numbers and proximity of recombination events. This can be overcome by incorporating more generations of recombination, for example an advanced intercross from the F2. Recombinant inbred lines (RIL) by selfing or full sib mating of a two-way cross also allow more accumulation of recombination. Also importantly, the lines so obtained are stable and therefore data can be collected over repeated generations and in different laboratories, enabling a large number of specialised traits to be analysed on the same stock. Thus the collaborative cross in the mouse was founded from eight inbred lines initially interbred to form an equal 8-way cross and from which RIL have been developed ([35]; http://mouse.ornl.gov/projects/collabcross.html). As the founder inbred lines have been densely mapped, genomic regions can be traced back to progenitor lines and many generations of recombination prior to fixation. In contrast an alternative mouse resource has been developed based on maintaining a heterozgygous closed stock; but, as it was also founded from crosses of inbred lines, recombinants can be traced back to small regions [36].

There have been many RIL established in plants. For example in Arabidopsis an advanced intercross was undertaken before undertaking the inbreeding to increase the opportunity for recombination [37]. In maize the NAM RIR lines were founded from crosses of 25 diverse inbred lines to a common reference parent [38], and together these represent a broad based population for analysis. 

### Genome Wide Association Studies (GWAS)

Linkage disequilibrium (LD) in a population reflects many generations of formation by drift and loss by recombination and the consequent association between markers and QTL has the potential for much finer mapping than do linkage studies, indeed for finding the causal genes or mutations and the responsible nucleotide difference (QTN). LD is minimised by sampling from the species as a whole or from large populations within it. Indeed for humans there is no opportunity to make specific populations and in the study of disease it is necessary to collect large numbers of affected individuals. Genome wide association studies (GWAS) have therefore become the method of choice and have been highly successful in identifying QTL in many species of animals and plants, with particularly intensive study of height and disease susceptibility in humans. Because many GWAS studies comprise samples from the population as a whole, they reflect natural genetic variation in quantitative traits such that inferences can be drawn about its architecture.

The power of GWAS is limited by numbers of individuals on which records are available, on the marker density, and on the rate at which LD diminishes with map distance. As in all QTL mapping approaches, power also depends on the size of QTL effects relative to the environmental variation, so for lowly heritable traits it is harder to detect QTL contributing the same proportion of the genetic variation. Studies are most efficient if a large number of traits can be recorded on the same individuals, for example by treating ‘affecteds’ for one disease as ‘controls’ for many others, and the data on height come as a by-product of disease studies. Precision is increased as more SNP markers are used, ultimately with complete sequence, which is necessary if LD falls very rapidly with map distance as in *D. melanogaster*. In dairy cattle, for example, the samples may comprise progeny tested bulls and their genotypes are analysed along with progeny mean phenotype, with data being collected in breeding programmes to utilise genomic selection (see below). In mice it has been suggested that commercially available outbred strains are suitable for GWAS analysis [39], and some specially constructed populations are also suitable for GWAS studies, such as those in mice [36] and maize [38].

In species where lab stocks can be maintained, an alternative is to establish lines from individuals sampled from the source population. Mackay and colleagues have therefore established the Drosophila Genetic Reference Panel using 192 iso-female lines (i.e. an inbred line formed from a single family) and complete genome sequencing of the line can be undertaken (http://service004.hpc.ncsu.edu/mackay/Good_Mackay_site/DBRP.html). Similarly humans are also being fully sequenced in the human genome project, and if phenotypic data on them are collected these too will provide information through GWAS. 

## THE GENETIC ARCHITECTURE OF QUANTITATIVE TRAITS – FINDINGS 

The extensive effort devoted to mapping has led to the identification of many QTL in many species and, in some cases, to the identification of the gene and the lesion. For example, in June 2011, 4682 QTL were listed on 376 different traits from 274 publications for cattle (http://www.animalgenome.org/cgi-bin/QTLdb/BT/index), 1747 were listed for maize, and 8646 for rice (http://www.gramene.org/db/qtl/qtl_display). These QTL are not necessarily all unique, however, and some so poorly mapped or comprising multiple QTL as to be of no practical value. Some of the many genes subsequently identified are of major importance. To take just a single trait, muscle development, the myostatin gene has impact in livestock and humans, including ‘double muscling’ in cattle [40], and the callipyge gene in sheep has led to the uncovering of new pathways for gene action [41].

In QTL mapping studies there is a considerable risk of detecting false positives, particularly if significance tests are done at low stringency. To keep the risk of type I errors to low levels requires very big data sets, and hence those used in GWAS studies have become increasingly large, often employing metapopulation analyses. These do not guarantee that the detected effect is localised to a single region, but may be influenced by effects at one or more others in high LD. Even so, proof of existence of a QTL requires cross validation, by replication in independent samples or, better, populations, and ultimately by direct identification of the genetic lesion concerned and showing its effect directly in a prospective study. 

Much of the effort has been expended in identifying individual QTL in crops and livestock with a view to utilising them in breeding programmes by marker assisted introgression or selection. In view of the greater importance of inbreds and their crosses in crop plants than farm livestock, it is not surprising that the techniques have been much more important in the former, albeit not having fulfilled all the initial optimism for improvement programmes in either plants [42, 43]) or animals [44]. 

The shape of the distribution of trait gene effects impacts on the number likely to be identified and their contribution to genetic variation or disease susceptibility. If few with large effects contribute much of the variation, it is both easy to detect them (high power, located far apart on the genome) and to utilise them effectively. Otherwise the tasks are harder, and become increasingly so the more genes that are involved: not only are genes/QTL of small effects missed, increasingly so as significance levels are raised as more markers are used in whole genome studies, but the effects of those detected may be overestimated (‘Beavis effect’). Rather than asking how many genes affect a trait, it is arguably more meaningful to assume that all genes affect all traits and the relevant unknown is the distribution of their effects. It is then reasonable to assume the shape is such that there are increasingly few of increasingly large effect, and then try to estimate this rate of decline and how long is the tail, despite knowing only a small segment of the distribution. There is some information on distributions of mutant effects in laboratory species [45], but that on segregating populations has, until very recently, been limited. A critical problem is to have a sufficiently powerful design that information can be obtained on QTL with effects of fractions of a standard deviation on the trait or that contribute well under 1% of the variance. Therefore the ability to distinguish between different long tail distributions, for example symmetric ones such as the reflected gamma, t or a mixture of normals is very poor. The large GWAS studies being undertaken with very dense SNP panels are providing some new insights, and I shall concentrate on these. 

### Findings from GWAS

The most comprehensive published GWAS data on a quantitative trait are for human height. From a metapopulation analyses of data from studies comprising over 180000 individuals, 180 QTL for height have been identified, each with high statistical stringency (P < 5 ×10^-8^) [46]. Many had previously been identified in more than one independent study. None of the SNPs individually accounts for more than 0.11% of the phenotypic variance in height and the estimated homozygote differences are typically under 1cm, compared to a phenotypic SD of about 7cm. To clarify, the variance contributed is that associated with the SNPs in LD with the gene or genes; this may be an underestimate of actual variance contributed because of imperfect LD or an overestimate in that it may account for multiple contributing sites. Although the heritability estimated from analyses of data on relatives is about 80%, together the 180 loci account for only just over 10% of the variance. Even if all unidentified common variants of similar effect sizes were identified, the authors estimate they would increase this figure to only about 16% [46, 47]. Of the QTL found, several (more than expected at random) were associated with genes characterized by abnormal skeletal growth. Several loci were identified which, on the basis of expression and other studies, are also strong candidates for growth genes. Thus it is reasonable to assume that all or most are in or near real trait genes. When SNPs near orthologous genes were tested in a cattle population, significantly more were associated with stature than would be expected by chance, indicative of common effects across species [48].

These results are closely mirrored by extensive studies of flowering time in maize. Using almost one million plants from a set of 5000 RIL from the NAM population (see above), Buckler and colleagues [38] found no evidence of any QTL of large effect, but many of smaller effects shared among families, with no substantial epistatic interactions. They note, however, that these results differ from those found in Arabidopsis and rice, both naturally self pollinated.

The extensive GWAS studies for many human complex diseases or other quantitative traits have identified contributing QTL but, despite using data sets of thousands of individuals, as for height all have failed to account for a high proportion of variance in the trait. This has sparked off a highly publicised debate on where is the ‘missing heritability’ [49]. Several explanations have been proposed [e.g. 50], typically related to the contributor’s expertise. These include rare variants, structural variants (e.g. duplications/deletions), epistatic effects, parent-of-origin effects, rare variants, transgenerational effects, epigenetic effects and biases in the estimate of the overall heritability. 

The high estimate of heritability for height, for example, comes consistently from different kinds of studies (e.g. see above using genomic relationship). None of the other potential causes can be ruled out, and rare variants with a large effect in trait units contribute little to the segregating variance. Nevertheless the basic explanation seems a simple one: that the GWAS studies have not yet captured most of the variation because there are numerous QTL of increasingly small effect or extreme frequency such that they are not detected with the SNP panels used at the power levels available in stringent significance tests. 

This is directly confirmed by GWAS studies undertaken by Yang, Goddard, Visscher and colleagues [51,52]. By taking densely mapped individuals which were not closely related to minimise environmental confounding and effects not directly associated with fitted SNPs, they assessed the amount of variation accounted for by all SNPs (over 580000 in the larger study [52]) without regard to whether or not they were statistically significant. Some 45% of the variation in height is accounted for, i.e. four times that by the ‘top’ 180 loci already identified [46]. It seems reasonable to assume that the remaining half or so of the genetic variance is due to QTL which are poorly marked, having insufficient LD with the SNPs. Similar analyses of body mass index and two metabolic measures [52] and of individuals affected by schizophrenia [53] showed that, although individual QTL accounting for only a small proportion of variance have been identified, fitting multiple SNPs simultaneously accounts for much more. Using a population in which there more highly related individuals, including some parents and sibs, almost all the genetic variation in height was accounted for by fitting SNPs [54]. In this analysis the SNPs were also, in effect, establishing the pedigree relationships and therefore LD well outside that associated with individual markers in the population as a whole.

The contributions of each chromosome can also be obtained in the GWAS analysis fitting all SNPs. This showed that the contribution for height is closely related to the length of the individual chromosomes [52] and therefore corresponds with an earlier analysis in which variation within full sib families was analysed [30]. These studies also showed a linear relationship between chromosome length and the number of individual significant loci for height discovered in GWAS analyses, together indicating a fairly random scatter across the chromosomes of genes affecting height. 

## EXPLAINING VARIATION WITHIN POPULATIONS 

### Population Genetics Background

The simplest model for maintenance of variation is a balance between genetic drift and mutation; although an oversimplification it is a useful reference point. The frequency density of alleles (ancestral or mutants) is then proportional to 1/[*p*(1 – *p*)], i.e. U-shaped. Assuming two alleles at a locus, the heterozygosity and genetic variance for an additive gene are proportional to *p*(1 – *p*), and therefore they have a uniform distribution over the allele frequency range from 0 to 1. Most mutations affecting a trait are likely to be deleterious with respect to fitness, either through pleiotropic effects on other traits or directly on that trait, for example if there is an intermediate optimum, and consequently they are usually lost quickly from the population [55]. Hence their frequency distribution is likely to be more extreme than for neutral mutants and the distribution of heterozygosity and thus variance for quantitative traits is also likely to tend to be U shaped. These predictions have several practical consequences in quantitative genetics both in QTL detection and in partition of variation.

Typically, a high proportion of the genetic variance obtained in conventional partitions of phenotypic variance is additive, and for abdominal bristle number in *D. melanogaster*, for example, it is essentially all additive (reviewed [56]). This does *not* imply, however, that the gene action is additive. If the gene frequency distribution is U-shaped, at most loci one genotype is likely to be so infrequent that almost all the genotypic variance is accounted for by the additive variance, whatever the degree of dominance. Similarly for pairs of loci, where only three genotypes, e.g. AABB, AABb and AaBB, are likely to be frequent if the a and b alleles are rare, epistasis can contribute little of the variance [56]. This is essentially a statistical rather than biological argument. Hence the knowledge that many major genes are known to be dominant and the findings of substantial epistasis in some QTL mapping experiments based on inbred crosses (review [57]) are not incompatible with the high proportions of additive genetic variance typically found from analysis of resemblance among relatives in segregating populations.

In GWAS, power of detection of a QTL with effect *a* on the trait is proportional to *r*^2^*a*^2^, where *r*^2^ is the squared LD correlation between QTL and marker alleles. The expected value of *r*^2^ depends not only on the closeness of the two but on their relative frequencies. Thus if a high proportion of trait genes have lower minor allele frequency than do the SNPs, many trait QTL are likely to be missed in GWAS studies, whether they are oriented at finding individual QTL [46] or fitting all SNPs to account for the variance [51, 52]. Thus increasingly dense marker panels with wider SNP frequency distributions should enable rather more variation to be detected. Detection of non-additive gene action in QTL analysis is less powerful than for additivity because dominance requires demonstrating a non-linear regression and two locus epistasis a two factor interaction, and it depends on higher order terms than *r*^2^. 

### Analysis and Implications of Continued Selection Response 

Artificial selection in a closed population can lead to long continued response. Notably, the Illinois maize selection experiment for high and low oil content in the kernel has continued for over 100 generations (= years) and, although low lines have reached plateaux (at almost 0% oil), the high lines have continued to respond and, for example, there was abundant variation present around generation 50 as evidenced by responses to reverse selection [58]. Such continuing responses have been seen in other experiments [59]. These results indicate that many genes must be affecting the trait, initially segregating and perhaps subsequently others arising by mutation, for with few loci contributing to the variation fixation would occur and response be attenuated. Linkage analysis of an advanced intercross (to reduce LD) of high and low lines made at generation 70 revealed that at least 50 QTL contributed to the divergence in oil content, that there was a strong correlation between their effects in pure line and crosses, and none of the effects exceeded 2% of the divergence [60]. 

With the availability of dense markers it is possible to track changes in frequency and selective sweeps, albeit distinguishing selection and drift is not simple. Johansson *et al*. [61] analysed long term broiler weight selected lines and found divergences between them at over 100 regions of the genome, a large proportion of which were likely selective. Previous analyses of F2 crosses of the same high and low lines had revealed some epistatic QTL with large effect, but analysis of the response indicates many loci were involved. It has been suggested that selection lines in breeding programmes of plants should be regularly monitored for marker gene frequency changes to identify associated QTL [62].

The indications are that broiler poultry still retain considerable variation as heritability for body weights remain around their ‘traditional’ 25%, despite over 50 generations of intensive and effective selection for growth and evidence from SNP analyses that variation was lost from native populations in the early periods of domestication prior to that [63]. These results also point to a highly polygenic architecture. 

Unsurprisingly, in view of the long continued responses to selection, we find from the GWAS and selection response analyses based not just on F2s that a very large number of genes are influencing the trait. Further, these studies do not detect substantial amounts of non-additivity and we do not expect much non-additive variance in any case in segregating populations [56]. Whilst this might be disappointing for those looking for genes of large effect, perhaps emboldened by initial F2 linkage analyses where significant effects are expected to be overestimated, for quantitative geneticists and breeders it is also nice to know that the multi-locus models they have used may be adequate representations of the real world. Hence it is not surprising that the infinitesimal model usually (but not invariably) does quite a good job of describing data and predicting breeding outcomes over multiple generations [64]. For example, the model fitted well to results from 20 generations of a selection experiment in mice for a measure of fat content where a four fold divergence was obtained between high and low lines [65], although that was not consistent across all other lines selected for different traits. From a plot of long term (50 generation) vs. first generation response for experiments in *D. melanogaster*, an infinitesimal model fitted almost as well as those developed with multiple loci of varying effects under models of maintenance of genetic variance, in each case taking account of selection in generating linkage disequilibrium [66]. This indicates that the pattern of selection response is not very informative about the architecture. An understanding of what underlying genetic changes have contributed to the change in the selected trait or traits requires an analysis at the molecular level. In view of the positive linkage disequilibrium between lines generated by selection among contributing QTL, however, interpretation of e.g. linkage mapping experiments among high selected *vs*. low or control lines lacks power [64], unless for example, many generations of recombination are first incorporated [e.g. 60]. Analysis of changes in frequency during selection or of selective sweeps [e.g. 61] is likely to be more informative.

### Pleiotropy

QTL mapping using line crosses is not a definitive method to detect pleiotropy as it is difficult to disentangle it from close linkage of genes each affecting only one of the traits. GWAS is rather more definitive in that much smaller pieces of the genome can be isolated and with some degree of certainty pleiotropic effects detected. A more direct approach is *via *mutation of individual genes, with insertional mutagenesis most convenient for subsequent analysis because the genetic lesion can be detected directly. 

Many studies have been undertaken in *D. melanogaster* by Mackay, who reported [e.g. 31] that of the P element insertion mutations screened, 22% affected abdominal bristle number, 23% sternopleural bristle number, 41% starvation stress resistance, 5.6% olfactory behaviour, 22% wing shape, 37% locomotor startle response, and 35% aggressive behaviour. These indicate substantial pleiotropy. 

In contrast, in two large studies of mutants, less pleiotropy was found [67]. In one, 253 morphological traits were recorded in each of 2449 haploid lines of *S. cerevisiae* mutant for a different gene and the mean number of traits affected in each line was 21.6 and the median was 7. In another, for 4905 genes and 308 traits in mice, the mean was 8.2 and median 8. Thus Wagner and Zhang [68] concluded that pleiotropy was limited. As high thresholds were set to avoid false positives, however, the chance of false negatives seems high and some of these results could be obtained even if all genes affected all traits, to varying but correlated extent. In view of the vast potential number of traits and high genetic correlations found among many of them, pleiotropy must be widespread and it would seem better, at least in principle if hard in practice, to fit and to validate models incorporating a joint distribution of effects of each gene on each trait. 

### Maintenance of Variation in Quantitative Traits

Whilst applications of genomics have demonstrated clearly that many loci contribute to variation in quantitative traits, they have not yet resolved one of the so far intractable problems: explaining the magnitude of variation seen in natural or domesticated populations. For example the coefficient of variation is typically around 10% for juvenile growth rate, lower for mature size, and higher for reproductive rate. Heritability is typically 25% for juvenile growth rate, higher for mature size and lower for reproductive rate. It is simplistic to argue that the heritability of fitness associated traits such as reproductive rate is low as it is under stronger natural selection, because the CV of such traits is high, such that a standardised measure, the genetic coefficient of variation (evolvability), shows a much narrower range.

Although there has been extensive analysis and discussion of what determines the level of genetic variation in populations (and a more limited one on what determines levels of the environmental variance, which itself must also be under genetic control), there is no clear resolution. There must be a trade-off between gain of variation from mutation (which adds a new heritability of typically 0.1% per generation) and loss by genetic drift and by most modes of selection. Basically it is hard to explain why levels of both genetic and environmental variation are as high as they are [3, 7, 17, 66, 69, 70]. The challenge is therefore to work out how the detailed genomic and other data becoming available can be used to address such basic problems in quantitative genetic problems. 

## ANIMAL AND PLANT IMPROVEMENT; PREDICTION OF DISEASE RISK

The availability of dense genomic markers is revolutionising the methods being used in animal breeding and is increasingly so in plant breeding and in disease prediction in humans. The principles and methods for ‘Genomic selection’ were proposed in 2001 for livestock by Meuwissen, Hayes and Goddard [71]. The basic idea is to fit all the markers and assume they are associated through LD with a random effect on the trait, sampled from defined distribution(s). Thus, for example, young dairy bulls or cockerels that have no phenotype for milk or egg production respectively from the same full sib family can be differentiated and extra information added on comparisons of animals in different families. This enables increased accuracy of selection and reduction in or elimination of progeny testing with reduced generation interval, according to the situation relevant to the breeding system and structure. Similarly the risk of some complex genetic disease of a young individual or foetus can be predicted from the incidences in and genetic similarity to adult relatives.

One simple view is to regard the predictor as simply that of improving estimation of the weighted proportion of genome or actual relationship (GBLUP) shared by relatives, which is equivalent to assuming effects of all SNPs are sampled from the same distribution, in essence an infinitesimal model (with linkage). The alternative proposed by Meuwissen *et al*. [71] is to assume some loci have larger effects than others, undertaken by assuming a mixture distribution of marker associated effects, or no effect, i.e. zero variance. This topic has generated extensive discussion and analysis [e.g. 72, 73]. GBLUP is conservative, in that it treats all regions of the genome equally, and has been adopted in dairy cattle evaluation in the USA, for example [74]. 

An essential component of assessment of methods of genomic prediction is some form of cross validation, whereby predictions of breeding values from a training set are checked by realisations on individuals which were not included in the initial predictions, the validation set. For example data from the most recent years can be excluded from the analyses leading to the predictions. 

Comparisons of accuracies of prediction under different models indicate that for some traits, such as proportion of white in the cattle coat, these are highest if there are assumed to be a relatively limited number of important genomic regions, whereas for milk yield GBLUP is accurate, suggesting more dispersed variation [75]. The accuracy of selection depends on the degree of LD between the QTL and the markers. It is therefore likely to be most accurate within populations (breeds) that are closed and of limited effective size, and less so when dealing with population mixtures or across generations, for example, as has been demonstrated [73]. Accuracy can be improved by increasing SNP panel size, ultimately by complete sequencing. Technological developments have been such that the costs of genome sequencing, for example, have reduced dramatically, whilst that of obtaining phenotypic data has not. The latter may well become limiting in the future. 

An alternative to genomic prediction fitting an additive quantitative genetic model or, exceptionally, dominance also within a Mendelian framework is to use non- or semi-parametric methods. With these any degree of epistatic interactions associated with the SNP marked effects can be fitted. Additive relationships can also be included for infinitesimal effects as in the usual model, but with a reproducing kernel Hilbert space regression fitted to the SNP associated affects [76]. At its simplest, consider selection of sires to breed reduced disease incidence. The training set of data comprises a group of genotyped sires each with a number of progeny, and SNPs are simply fitted without, for example, incorporating relationships [77]. The function of the SNPs giving the highest accuracy of prediction using this training set is then chosen. It can then be tested by cross validation. A feature of this approach is that it incorporates all possible epistatic interactions (up to the order of number of SNPs) and avoids assumptions about the inheritance mechanisms. Predictions incorporating these non-additive effects from the markers are not transferable across generations, however. 

Incorporation of genomic prediction has the potential to greatly increase rates of genetic improvement of livestock and it is rapidly being taken up [73, 74, 78]). Essentially the same methodology can be and is being incorporated into plant breeding programmes, depending on the breeding system [79-80]. Prediction of risk of disease in individual humans can be tackled by the same approach [8, 81, 82]. 

## CONCLUDING REMARKS

Our understanding of quantitative genetic inheritance has largely been at the phenotypic level, summarised by variances and covariances using increasingly sophisticated statistical and computing methods. Developments continue in this area, notably in previously less well studied areas, such as of natural populations. These methods have been used successfully in effecting genetic change in livestock and plants. As QTL mapping has progressed with increasingly dense markers available with genomic techniques, now feasible using LD based genome wide association studies, so has information about the effects on the traits of individual loci accumulated and been utilised in marker assisted introgression and selection, to some extent in animal breeding and more in plants. These studies have, however, confirmed the multi-locus nature of quantitative genetic variation, previously largely inferred but not proven from selection and other experiments. At this stage genomic methods are therefore having their most impact in breeding practice in genomic prediction, expanding what is essentially a statistical approach. In view of the complexity of the genome and the evidence, unsurprising to most quantitative geneticists, that many genes contribute to variation, we must appreciate that exact descriptions even at the level of numbers of genes, distributions of gene effects and their interactions will be very hard to resolve. Fortunately, however, we can still make progress without all that detailed knowledge by introducing extra information from variation at the genome level into our descriptions and predictions.

I have not dealt with the area here, but the hope and expectation is that techniques from genomics, transcriptomics and other ‘omics will increase our knowledge of how the genes that affect quantitative traits act in the organism to do so, and how they are regulated. Indeed these are likely to be informed by quantitative genetic approaches: for example transcript abundance is itself a quantitative trait. There is a hope that systems biological approaches will be fruitful in unravelling the chain from gene to phenotype. It seems likely that understanding will come first from the study of complex disease where understanding individual genes is a priority and it will probably be a slow process for continuous traits, for which only a few pathways are unlikely to predominate. In due course this will help us not only to make better predictions in health and breeding but also to understand more about how our species have evolved. We now have extensive information at the genomic level on species differences for example, but little on how the differences in quantitative measures such as size, longevity and behaviour have arisen. There is plenty to do.

We now have the ability to record genomic sequences, obtain levels of expression of all genes in different environmental circumstances and manipulate the vast amounts of detail so as to construct pathways of gene action and interaction. The problem is how this information should be put together, both to understand how the system works and to use it to our benefit in improvement of food production, food quality, health, and the environment. 

Traditionally, and perhaps inevitably in view of the need to justify research grants, there has been long running optimism about understanding variation in quantitative traits. Much of it is still to be realised The quantitative nature of the traits and the polygenic influence on them will make this a very challenging task.
